# Peristomal bullous pemphigoid: expanding the spectrum of localized presentations^☆^

**DOI:** 10.1016/j.abd.2026.501390

**Published:** 2026-06-16

**Authors:** Fernanda Ramírez-Bravo, Catalina Arbat Contreras, Gonzalo Hevia Marcet, Isidora Wajner Martínez, María Jesús Urrutia, Alex Castro

**Affiliations:** a Department of Dermatology, Faculty of Medicine, Clinica Alemana, Universidad del Desarrollo, Santiago, Chile; b Department of Dermatology, Padre Hurtado Hospital, Santiago, Chile; c Faculty of Medicine, Universidad de Los Andes, Santiago, Chile; d Department of Pathology, Faculty of Medicine, Clinica Alemana, Universidad del Desarrollo, Santiago, Chile; e Faculty of medical Sciences, Universidad de Santiago, Santiago, Chile

Dear Editor,

Peristomal Bullous Pemphigoid (BP) is an exceedingly rare variant of localized BP. Due to its uncommon presentation, these cases are often misdiagnosed because of their clinical resemblance to more common dermatoses in this region, such as irritant and allergic contact dermatitis, bacterial and viral infections, including impetigo, herpes simplex, dermatitis herpetiformis, and pyoderma gangrenosum, implying significant diagnostic challenges.^1^

We present a case of an 83-year-old male with a history of surgically treated colorectal cancer (CRC) and a permanent colostomy. Three months post-operation, the patient developed tense blisters initially localized around the stoma, which progressively extended to the right flank and periumbilical region. He reported no prior similar skin involvement elsewhere. Multiple courses of systemic antibiotics were administered for suspected bullous impetigo, with no clinical improvement. Physical examination revealed a tense blister adjacent to the right side of the stoma, accompanied by erosions, extending to the right flank and periumbilical region (Fig. 1), without mucosal involvement. Given the atypical presentation and lack of response to antibiotics, localized bullous pemphigoid was suspected. Two skin punch biopsies were obtained: one from perilesional skin for Direct Immunofluorescence (DIF) and another from the edge of the blister, including adjacent healthy skin, for Hematoxylin & eosin (H&E) staining.

**Figure 1:**
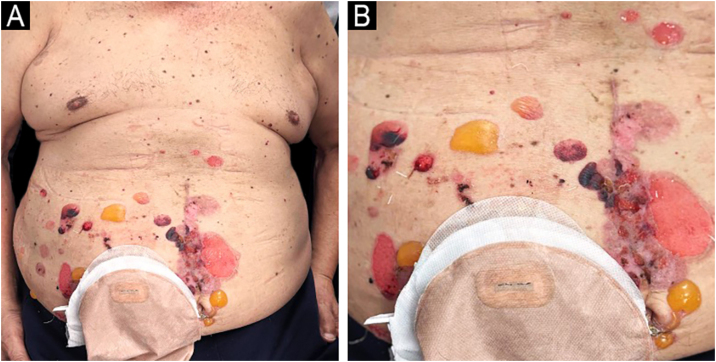
Clinical presentation of peristomal bullous pemphigoid. (A) Multiple tense and flaccid bullae, erosions, and crusted lesions predominantly around the stoma, with scattered lesions over the abdomen and chest. (B) Close-up showing grouped yellowish and hemorrhagic bullae, erosions, and crusts on an erythematous base surrounding the colostomy site.

Histopathological examination (Fig. 2) demonstrated a subepidermal blister with a mixed inflammatory infiltrate rich in eosinophils. The adjacent epidermis showed vacuolar degeneration of the basal layer and mild spongiosis. The papillary dermis exhibited edema and a perivascular and interstitial infiltrate predominantly composed of eosinophils. Periodic Acid-Schiff (PAS) staining was positive at the floor of the blister, confirming that the separation occurs above the lamina densa. DIF revealed linear deposits of C3 and weak IgG along the Basement Membrane Zone (BMZ), suggesting the diagnosis of bullous pemphigoid. Serological analysis further confirmed the diagnosis, yielding positive ELISA results (EUROIMMUN, Lübeck, Germany) for anti-BP180 (Ratio > 1.0; cutoff ≥ 1.0) and anti-BP230 (28 UR/mL; cutoff ≥ 20 UR/mL) autoantibodies. High-potency topical corticosteroids, the mainstay of treatment for localized BP, were initiated with a complete response.

**Figure 2:**
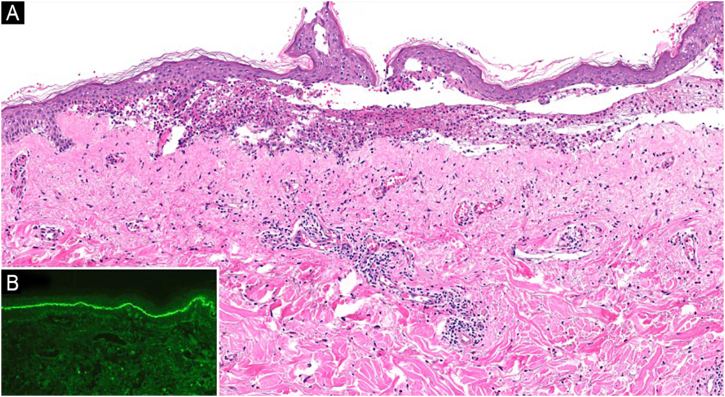
Histological images. (A) Hematoxylin & eosin 200×. Subepidermal blister containing fibrin, lymphocytes, some neutrophils and numerous eosinophils. (B) DIF 200×. Linear C3 deposits can be seen along the basal membrane zone.

Localized peristomal BP is a rare and underreported condition, with only 10 published articles that report a total of 27 patients to date, 11 of whom exhibited disease exclusively localized to the ostomy site. In these cases, malignant neoplasia was the main cause of ostomy, followed by Inflammatory Bowel Disease (IBD).

The underlying pathophysiology is not yet fully understood, but it is believed to involve an interaction between genetic predisposition (pre-existent serum antibodies against the BMZ) and local triggering factors such as surgery. Tissue injury and wound remodeling post-surgery may increase vascular permeability via Vascular Endothelial Growth Factor (VEGF) synthesis, facilitating the migration of granulocytes and other inflammatory cells and promoting elevations in circulating anti-BP180 and anti-BP230 levels.

Finally, the complement system is activated by the binding of granulocytes to anti-BMZ autoantibodies, triggering blister formation.^2^ Since BP is caused by autoantibodies directed against two hemidesmosomal antigens, BP180 and BP230,^3^, ^4^ these molecules play a key role in the pathogenic process and thus contribute to diagnostic confirmation.

Alternatively, the epitope spreading theory suggests that an immune response is initiated by the release of antigens during chronic immune or inflammatory processes. In this context, plectin (a cytoskeletal protein structurally similar to BP230 and present in both intestinal mucosa and cutaneous cells)^5^ may serve as the initiating epitope. Notably, plectin’s cell membrane expression is increased in colonic tissue with active inflammation, such as in invasive CRC and IBD. Autoantibodies produced against plectin may subsequently cross-react with cutaneous plectin or BP230, resulting in the clinical manifestations characteristic of localized BP.^5^

Furthermore, the quantitative determination of anti-BP180 IgG levels by ELISA is essential for monitoring clinical disease activity and guiding therapeutic decisions.^6^ Low or negative anti-BP180 values in patients achieving remission carry a high negative predictive value (approximately 90%), making follow-up testing advisable prior to treatment discontinuation.^4^

This case underscores the importance of maintaining a high index of suspicion for bullous pemphigoid in the differential diagnosis of peristomal skin lesions, particularly in elderly patients with a history of colorectal cancer or IBD, and highlights the need to integrate clinical, histopathological, and immunopathological data to ensure timely and accurate diagnosis and management.

## ORCID IDs

Catalina Arbat Contreras: 0009-0009-1166-4797

Gonzalo Hevia Marcet: 0000-0001-5514-064X

Isidora Wajner Martínez: 0009-0001-9773-9009

María Jesús Urrutia: 0009-0000-3470-5904

Alex Castro: 0000-0003-4431-5293

## Research data availability

Does not apply.

## Financial support

None declared.

## Authors' contributions

Fernanda Ramírez-Bravo: Critical review of the literature; critical review of the manuscript; approval of the final version of the manuscript.

Catalina Arbat Contreras: Critical review of the literature; critical review of the manuscript; approval of the final version of the manuscript.

Gonzalo Hevia Marcet: Critical review of the literature; critical review of the manuscript; approval of the final version of the manuscript.

Isidora Wajner Martínez: Critical review of the literature; critical review of the manuscript; approval of the final version of the manuscript.

María Jesús Urrutia: Critical review of the literature; critical review of the manuscript; approval of the final version of the manuscript.

Alex Castro: Critical review of the literature; critical review of the manuscript; approval of the final version of the manuscript.

## Conflicts of interest

None declared.
